# Observation and Measurement of Negative Differential Resistance on PtSi Schottky Junctions on Porous Silicon

**DOI:** 10.3390/s100201012

**Published:** 2010-01-27

**Authors:** Seyedeh Maryam Banihashemian, Hassan Hajghassem, Alireza Erfanian, Majidreza Aliahmadi, Mansor Mohtashamifar, Seyed Mohamadhosein Mosakazemi

**Affiliations:** 1 Department of Physics, Islamic Azad University, Qom Branch, Qom, Iran; E-Mail: banihashemian_physics@yahoo.com; 2 Electrical Engineering Department, Shahid Beheshti University, Tehran, Iran; 3 Electrical Engineering Department, K.N. Toosi University, Tehran, Iran; E-Mail: erfanian@icee.ac.ir; 4 Electronic Research Center, Tehran, Iran; E-Mails: mr_aliahmadi@yahoo.com (M.A.); TAH1965_MOH@yahoo.com (M.M.); 5 Qom Payame Noor University, Iran; E-Mail: mh_moosakazemi@pnu.ac.ir

**Keywords:** nanoporous Si, platinum silicide, quantum tunneling effect, negative differential resistance

## Abstract

Nanosize porous Si is made by two step controlled etching of Si. The first etching step is carried on the Si surface and the second is performed after deposition of 75 Å of platinum on the formed surface. A platinum silicide structure with a size of less than 25 nm is formed on the porous Si surface, as measured with an Atomic Forced Microscope (AFM). Differential resistance curve as a function of voltage in 77 K and 100 K shows a negative differential resistance and indicates the effect of quantum tunneling. In general form, the ratio of maximum to minimum tunneling current (PVR) and the number of peaks in I–V curves reduces by increasing the temperature. However, due to accumulation of carriers behind the potential barrier and superposition of several peaks, it is observed that the PVR increases at 100 K and the maximum PVR at 100 K is 189.6.

## 1. Introduction

Devices which exhibit negative differential resistance are widely used in oscillators, photosensitive, memory, computing technology and microwave devices. In 1957, negative differential resistance (NDR) was first observed in Esaki tunnel diodes. These diodes have heavily doped junctions and when becomes forward bias, exhibit negative resistance at low voltages due to quantum-mechanical tunneling effects. Discrete energy level tunneling based NDR was also reported from several experimental systems such as quantum well structure, single electron devices and gun diodes [1,2]. Metal-insulator-metal (MIM) structure with nano scale dimensions can exhibit NDR as a result of quantum-mechanical tunneling effect [3–5].

In 2008 Fanga *et al.* demonstrated negative-differential-resistance (NDR) with high peak-to-valley current ratios (*i.e.*, larger than 5) in Al/Alq3 structures at room temperature [6]. In 2000 Gamage *et al.* reported that based on measurements in micro machined silicon flow sensors, the operation in the NDR regime results in increased sensitivity compared to operation in the saturation regime. The increased sensitivity in this regime occurs at relatively higher temperatures with a possible increase in power consumption [7]. Jiang *et al.* reported that a novel infrared sensitive (*i.e.*, SiC/Si) NDR device with excellent characteristics had been achieved at 100 °C [8].

In this work, the observation of NDR in an IR detector with non uniform array of nanoscale platinum silicide and nanosize porous silicon with a high peak-to-valley ratio in metal–semiconductor-metal (MSM) structure is investigated. This structure is made without using any lithography process. After porous Si is made by a controlled chemical etching process, 75 Å of platinum is deposited on the porous Si surface using e-beam evaporation. A second etching process is then followed using a lower concentration of HF. The heights of the Si walls in the porous structure are lowered to nearly 99 nm and their thicknesses are reduced to about 35 nm after the second etching process. The thickness and height of porous Si are measured by an Atomic Force Microscope (AFM) system.

There is a band bending in Pt and Si energy band diagram and a Schottky barrier is formed. Since the potential barrier between metal and semiconductor is much larger than a few KT, the main mechanism with which carriers can transfer to the other side is by thermionic emission. As a result, the current voltage relation is of the form of barrier type. The barrier height at the Pt and Si junction is 0.25 eV, which is suitable to detect IR signals (*i.e.*, see Figure 2). However, when porous Si is formed, its band gap energy increases and there will be a detection shift toward the higher frequency range. This frequency shift depends on the size of formed porous and can continue up to UV range for small porous sizes. The thickness of metal_Si interface barrier is about a few nanometers. The carrier tunneling to the other side can be improved by reducing this thickness and as a result the NDR can be observed in the I–V curve. dV/dI as a function of voltage measured at 77 and 100 K temperature show negative differential resistance. The ratio of maximum to minimum tunneling current (PVR) and the number of peaks in I–V curves reduces by increasing the temperature. However, due to accumulation of carriers behind the potential barrier and superposition of several peaks, it is seen that the PVR increases at 100 K and the maximum PVR at 100 K is 189.6. This negative differential resistance, as will be discussed in the following sections, can be explained by quantum tunneling effect. This array structure is behaving like an array of potential barriers and potential wells. Due to small thickness of Si walls and PtSi valleys, one can expect the density of energy states in small size of porous silicon to be quantized. Perhaps a possible way of explaining NDR in this structure is the formation of quantum dot array of nano platinum silicide on porous silicon. This of course needs to be further investigated.

## 2. Experimental Section

**Figure 1.**
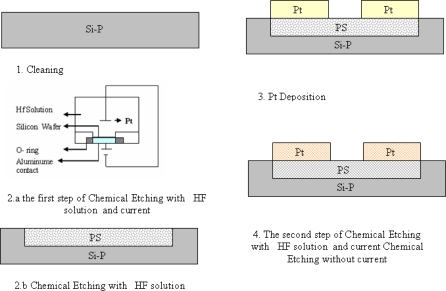

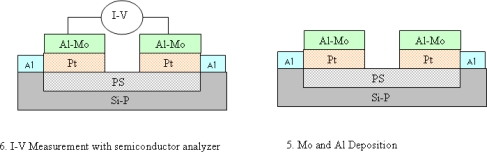
Schematic view of the sample. The device formation steps are shown in this figure. Four side squares (*i.e.*, Al and Al-Mo) in these figures are used for electronic contacts. Porous silicon (PS), platinum (Pt), silicon P type (Si-P).

**Figure 2.**
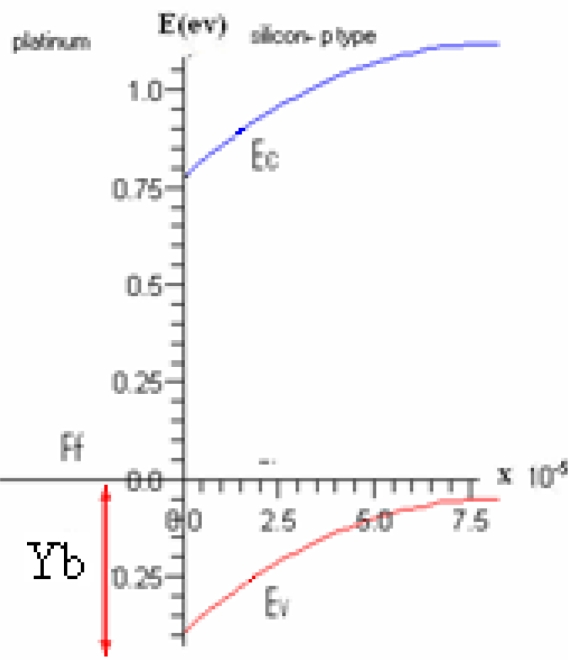
Energy band diagram in platinum—silicon p-type contact. Ef is Fermi surface in metal—semiconductor junction, Ec is conduction band, Ev is valance band, Yb is potential barrier in platinum-silicon contact (Y-axis) and X-axis is distance.

The starting material was P-type Si }100{. After RCA cleaning, electrochemical prosification is accomplished by using an electrolytic cell with 40% HF-C_2_H_5_OH-H_2_O solution with the ratio of 1:2:1 at constant current density of 50 mA for 15 minutes [16]. Seventy five Å of platinum is then deposited on the porous Si surface, using e-beam evaporation. The deposited layer was then annealed at 400 °C for 30 minutes. After the annealing process, the platinum on the porous Si surface produces non uniform platinum silicides. For a better control of the etching process a second etching was then performed using lower concentration of HF and with no dc current to etch the part of surface with less concentration of platinum silicides. As a result we obtained more nanoscale size etching in this step. To make contact to the silicide surface, 100 nm of molybdenum is deposited on the surface using e-beam evaporation (10^−5^–10^−6^ Torr). One hundred and seventy five nm of Al is then thermally evaporated on the formed surface followed by 20 minutes of annealing at 200 °C. The molybdenum is used as blocking layer for stopping the diffusion of Al into the underlying substrate (see Figure 1). After device fabrication, I–V measurements were made at 77, 100 and 300 K temperature using a HP4145B semiconductor parameter analyzer. Measurements were made on PtSi/PS/PtSi and PtSi/PS/PtSi/Mo/Al structures and both structures exhibit the same behavior. Since the Mo/Al is only used for ohmic contacts, instead of PtSi/PS/PtSi/Mo/Al the PtSi/PS/PtSi structure is addressed in this article. The formed patterns are inspected by the images obtained from Atomic Force Microscopy (AFM-Dualscope ™ DS 95 Series).

## 3. Results and Discussion

When platinum is deposited and annealed on the porous Si surface, it forms a new phase of material in the interface of platinum and Si. Each pPlatinum atom in porous Si is combined with a Si atom in a one to one ratio [9]. The platinum silicide is formed after annealing and an array of metal (*i.e.*, platinum silicide) and semiconductor (Si) with different band gap energy is constructed. While Si is consumed in the process of PtSi formation the thickness of porous walls start to shrink and as a result an array of nanosize PtSi is constructed. Low potential barrier PtSi made on p-type Si can detect infrared signals and the formation of nano array in this structure with NDR behavior can extend its applications.

**Figure 3.**
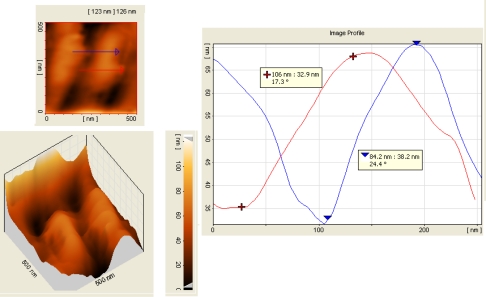
AFM image of porous Si before Pt deposition. The top left figure is two dimensional image in an area of 500 nm by 500 nm and the bottom left figure is its 3D surface image, where its analysis is shown in the right figure along the arrows.

**Figure 4.**
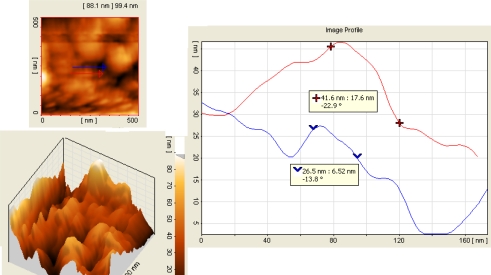
AFM image of porous Si after Pt deposition and second stage of etching. The top left figure is two dimensional image in an area of 500 nm by 500 nm and the bottom left figure is its 3D surface image, where its analysis is shown in the right figure along the arrows.

AFM images taken from a 500 nm by 500 nm samples in this structure are shown in Figures 3 and 4. The curve in the right side of these figures show the height of the porous wall in the Y axis as a function of its thickness for a side sliced view of the porous Si nanoscale platinum silicide and nano- porous silicon in metal–semiconductor–metal (MSM) structure forms double potential barrier. MSM structures consist of a semiconductor between two metals which forms a back-to-back Schottky contacts on the semi-insulating substrate. Due to the quantum confinement effect, the energy band gap of semiconductor nanostructure increases with decreasing the size of quantum structures [11]. As a result the band width of IR detection shifts to the shorter wave lengths.

Before platinum deposition and the second etching process, thin hills with an average width of 70 nm and average height of 126 nm are seen in these pictures. After platinum deposition and the second etching process, however, the width and height of hills are reduced to 34 nm and 41 nm, respectively, as it is shown in these pictures. Since each platinum atom is combined with one Si atom in a one to one ratio, therefore the formed PtSi film has nearly double thickness of platinum (*i.e.*, 15 nm = 7.5 × 2) [9,10 ].

**Figure 5.**
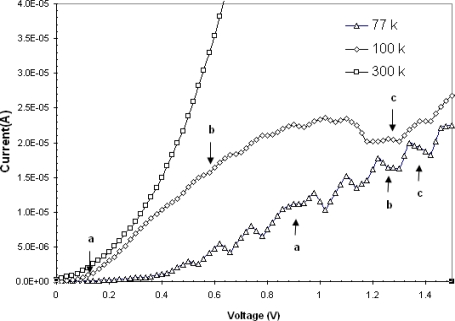
Comparison of I–V curves of PtSi/PS/PtSi structure at 77, 100 and 300 K temperature.

An MSM structure has some similarity with metal–semi insulator–metal (MSIM) structure and we can expect to observe double tunneling barrier behavior in MSM structure. In porous samples, the surface of junctions are irregular and in random directions. In nanosize pores the scale with which the direction changes are comparable to the mean free path of the carriers. This has an important effect on number of carriers transferred into the substrate. By depositing Pt on porous Si, it will also be deposited into these pores and after annealing the silicide will be grown over the walls [12]. As it was mentioned previously, when the size of a material such as metal or semiconductor is reduced to a few nanometers, its electronic structure will exhibit the discrete energy level due to quantum confinement effect. When energy levels in nanoscale materials are discrete [13–15], the resultant tunneling would be quantized and so peaks with negative differential resistance may be observed in their I–V curves. The energy level separation in small size materials (*i.e.*, about nanometer) can be estimated by the voltage difference between the peaks [16]. I–V measurements in PtSi/PS/PtSi structure are made at 77, 100 and 300 K temperature in this work.Few peaks are observed in 0 to 1.5 volts region at 77 K as shown in Figure 5.

**Figure 6.**
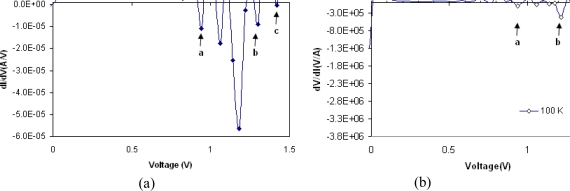
Differential conductance curve (dI/dV) as a function of voltage at 100 K (left figure). Differential resistance curve as a function of voltage at 100 K (right figure). Negative differential resistance can be seen at points a, b and c.

**Figure 7.**
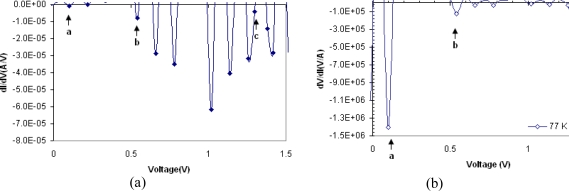
Differential conductance curve (dI/dV) as a function of voltage at 77 K (left figure). Differential resistance curve as a function of voltage at 77 K (right figure). Negative differential resistance can be seen at points a, b and c.

There is generally a relation between peak voltages and the discreet energy states in the valance and conduction bands. The current is maximum when the energy level in metal is aligned with a single energy level in semiconductor. On the other hand the current is at its lowest value when the energy level in metal is located between two energy levels. This explains the observed variation in current voltage curve. In this MSM structure the probability of tunneling is high. This high tunneling current can be seen in the peaks and negative differential resistance observed in the I–V curves. Therefore one possibility is that these peaks are caused by the electron tunneling through the quantized energy levels. Figure 5 shows that the number of peaks reduces by increasing the temperature. However due to parasitic resistance of contact points, a shift toward higher voltages is expected. Since the peaks positions are temperature dependent, and due to the measurements limitation, usually a dominant peak which is due to superposition of several other peaks is seen as shown in Figure 5 at 100 K. Figures 6(a) and 7(a) show the differential conductance (*i.e.*, dI/dV) as a function of voltage and Figures 6(b) and 7(b) show the differential resistance (*i.e.*, dV/dI) as a function of voltage, obtained from the I–V measurements made at 100 and 77 K respectively. The maximum negative differential resistances in Figures 6(b) and 7(b) are denoted by a, b and c marks.

At the points in Figures 6(a) and 7(a) where the negative differential conductance is at minimum value the negative differential resistance is maximum. The ratios of maximum and minimum currents at peaks (*i.e.*, the peak to valley ratio is PVR) for a, b and c points are shown in Table 1. As shown in this table, at low value of voltages, the PVR for points a and b decreases with temperature. However, at higher voltages due to accumulation of carriers behind the potential barrier and superposition of several peaks, it is seen that the PVR increases at 100 K and the maximum PVR at 100 K is 189.6. As it can be seen in Figure 5 the current is almost constant at point a at 77 K. This is due to accumulation of carriers behind the potential barrier. However, by increasing the voltage the conducting condition for carriers increases almost instantly and so we can observed a large negative differential resistance. At higher temperatures (*i.e.*, ∼300 K) no peak is observed. The ratio of maximum to minimum currents, due to the tunneling, should decrease by increasing the temperature By increasing the temperature the tunneling current due to several discrete energy states superimpose and their effects cancel out and no NDR is observed. Carriers with higher thermal energy on the other hand contribute to thermionic emission and it will be the dominating current at higher temperature. It can be inferred that at higher temperatures the variation in total current is more pronounced than the tunneling current variation.

## 4. Conclusions

Nanosize porous Si is made by two step control etching of Si when 75 Å of platinum is deposited on a Si surface. Quantum arrays of platinum silicide (*i.e.*, nanostructure of a three dimensional detector array) with sizes of less than 25 nm are formed on the porous Si surface measured with an Atomic Forced Microscope (AFM). Our results shows a nonlinear relation for carrier transport in I–V curve measured in this structure. Peaks with negative differential resistance are observed in the I–V measurements made at 77 K and 100 K respectively. The ratio of maximum to minimum tunneling currents (*i.e.*, PVR) and the number of peaks in I–V curves reduces by increasing the temperature. However, due to accumulation of carriers behind the potential barrier and superposition of several peaks, it is seen that the PVR increases at 100 K and the maximum PVR at 100 K is 189.6. According to the results obtained in this work we may be able to implement three dimensional IR detectors with a better quantum efficiency and larger detection band width using nanosize porous Si.

## Figures and Tables

**Table 1.** Tunneling rate at points a, b and c for temperature of 77 and 100 K.

**PVR/T(K)**	**T = 77 K**	**T = 100 K**
PVR a	72.9	2.1
PVR b	5.1	4.7
PVR c	22.5	189.6
